# Combined detection of COMP and CS846 biomarkers in experimental rat osteoarthritis: a potential approach for assessment and diagnosis of osteoarthritis

**DOI:** 10.1186/s13018-018-0938-3

**Published:** 2018-09-12

**Authors:** Tianwen Ma, Zhiheng Zhang, Xiaopeng Song, Hui Bai, Yue Li, Xinran Li, Jinghua Zhao, Yuanqiang Ma, Li Gao

**Affiliations:** Heilongjiang Key Laboratory for Laboratory Animals and Comparative Medicine, College of Veterinary Medicine, Northeast Agricultural University, Harbin, 150030 China

**Keywords:** Osteoarthritis, COMP, CS846, Serum, Biomarkers

## Abstract

**Background:**

To comprehensively evaluate the diagnostic value of serum cartilage oligomeric matrix protein (COMP) and chondroitin sulfate 846 epitope (CS846) biomarkers in osteoarthritis (OA), longitudinal and combined measurement of serum COMP and CS846 were performed at different stages in the pathological process of OA in a rat model of anterior cruciate ligament transection (ACLT).

**Methods:**

Sixty male Sprague-Dawley rats were randomly divided into two groups, including a model group (*n* = 30) and a control group (*n* = 30). Rat models were established by ACLT surgery, and sham operations were performed on rats in the control group. Prior to surgery and at 2, 4, 6, 8, and 10 weeks after ACLT surgery, serum levels of COMP and CS846 biomarkers were determined using an enzyme-linked immunosorbent assay approach. Five rats per group were euthanized at 2, 4, 6, 8, and 10 weeks after surgery, after which tibial plateau specimens were collected. Macroscopic observation and histological examination were employed for rat tibial plateau. Histological changes in articular cartilage were evaluated according to Osteoarthritis Research Society International (OARSI) scoring criteria. The area under the curve (AUC) of COMP, CS846, and combined biomarkers was compared using receiver operating characteristic (ROC) curve.

**Results:**

Within 10 weeks after surgery, serum levels of COMP and CS846 in the model group were significantly higher when compared to those in the control group. Moreover, a significant correlation was observed between changes in COMP and CS846 levels. At each time point, macroscopic observations and OARSI scores were significantly increased in the development of OA. The AUC of combined biomarkers was higher compared to that of COMP and CS846 alone. Finally, a positive relationship was found between levels of COMP and CS846 and the OARSI score.

**Conclusions:**

In this study, we found that combined detection of serum CS846 and COMP levels can be used for diagnosis and monitoring of OA progression.

## Background

Osteoarthritis (OA) is a progressive degenerative joint condition that is estimated to affect over 250 million individuals worldwide [1]. Currently, there is no effective cure to treat OA [2]. Presently, the diagnosis of OA is based on clinical symptoms and traditional radiology. Magnetic resonance imaging (MRI) not only assesses changes in cartilage during OA development, but also assesses joint tissue damage. However, due to financial constraints and the lack of an internationally validated assessment scale, the use of MRI in the routine diagnosis of OA is limited [3], and X-rays remain the routine diagnostic method. Although the accuracy and sensitivity of these approaches are relatively high, these methods fail to distinctively identify the developmental stages of OA. Similarly, most of the current OA treatments are largely palliative until the articular cartilage has been severely damaged; during the development of OA, the joints become completely dysfunctional and prosthetic replacement becomes necessary [4]. Therefore, an effective method for early diagnosis of OA is imperative, and early changes in OA may be reversed by effective therapeutic drugs.

OA is often not detected until the middle or end stage [5–7]. In recent years, OA biomarkers have received increased attention as an objective indicator for early diagnosis of OA and for assessment of the disease process [8]. OA biomarkers can be detected at an early stage of OA, prior to the presence of radiographic signs. Cartilage oligomeric matrix protein (COMP) is a non-collagenous component of cartilage, which accounts for roughly 1% of the wet weight of articular tissue [9]. COMP promotes the combination of type II collagen fibers and stable fiber networks. Chondroitin sulfate (CS) is a glycosaminoglycan (GAG) that is covalently attached to specific proteins to form proteoglycans, which are abundant components of the extracellular matrix (ECM) [10]. CS 846 epitope (CS846) is a CS synthetic marker and inseparable from the degree of joint injury in patients with OA. CS846 is a by-product of proteoglycan metabolism and has been detected in serum and synovial fluid (SF) [11]. Therefore, CS846 and COMP as sensitive and specific biomarkers reflecting degradation of cartilage and synovial tissues have increasingly been used as helpful promising tools in early OA diagnosis before irreversible damage has occurred.

However, in many studies of early OA diagnosis, only one biomarker in the pathological process of OA was measured at a single time point [12, 13]. In verifying our hypothesis that COMP and CS846 can be used as a joint biomarker for knee OA, a longitudinal study of the combined measurement of serum levels of COMP and CS846 was performed using an anterior cruciate ligament transection (ACLT) rat models in which the relationship between COMP and CS846 levels and the degradation of articular cartilage were evaluated. The area under the curve (AUC) of COMP, CS846, and combined biomarkers in the evaluation and diagnosis of osteoarthritis was analyzed by ROC curve analysis. When promising, combined biomarkers that were identified for clinical use may have a beneficial effect on both patient health and the medical economy.

## Methods

### Animals

A total of 60 male Sprague-Dawley rats (11–12 weeks old, 300–350 g) were purchased from the Animal Experimental Center of the Second Affiliated Hospital of the Harbin Medical University (Harbin, China) and housed in a controlled environment (light/dark, 12/12 h; temperature 23 ± 1 °C). Rats had access to water and food ad libitum. Ethical treatment of animals in this study was approved by the Animal Welfare Committee protocol (#NEAU-2017-02-0252-11) at Northeast Agricultural University (Harbin, China). All efforts were made to minimize animal suffering and to reduce the number of animals used.

### Surgically induced osteoarthritis in rats

Prior to use, animals were acclimated for 1 week. Then, rats were randomly assigned to the model group (*n* = 30) or control group (*n* = 30). Rats in the model group were anesthetized by inhalation of 2% isoflurane (catalog no. C008170801, Yipin Pharm-hebei, China) in oxygen/nitrous oxide, and the right knee was shaved and scrubbed to prepare for surgery. Rats underwent ACLT via an incision on the medial aspect of the right knee joint capsule, anterior to the medial collateral ligament. The anterior cruciate ligament (ACL) was transected using a microsurgical knife using a surgical microscope (Corder Optics ad electronics Co., Ltd., Chengdu, China). After irrigation with saline to remove tissue debris, the patella was relocated. Next, a positive anterior drawer test was performed to ensure complete transection of the ligament. The wound was closed with braided, absorbable polyglycolide absorbable suture 6/0 (Jinhuan Medical Products Co., Ltd., Shanghai, China). During surgery, close attention was paid not to damage the articular cartilage. No surgery was performed on the left hind knee. After surgery, rats were allowed to walk freely. Rats were administered intramuscular injections of penicillin (400,000 units) once daily for three consecutive days. Rats in the control group were sham-operated using the same approach but without any ligament transection or meniscectomy.

### Sample collection and storage

In this study, right knee joints and serum were harvested at 0, 2, 4, 6, 8, and 10 weeks after surgery (*n* = 5 rats per group). Animals were euthanized using an overdose of diethyl ether, and right knee joints were collected. Blood samples were obtained from the orbital vein, and serum was collected in Eppendorf tubes without additives. Next, blood was centrifuged at 1000×*g* for 20 min at room temperature. Supernatant was collected and stored at − 80 °C until future analysis.

### Macroscopic observation

For macroscopic observation, rat tibial plateaus were collected. Cartilage degradation on the surface of tibial plateau was evaluated using a dissecting microscope, and the degree of degradation was graded on a scale of 0–4 as follows: 0 = surface smooth with normal color; 1 = surface rough with minimal fibrillation or a slight yellowish color; 2 = cartilage erosion extending into superficial or middle layers; 3 = cartilage ulceration extending into deeper layers; and 4 = cartilage depletion with subchondral bone exposed [14]. Macroscopic observations were conducted by an observer who was blinded to the groups.

### Safranin O staining, OARSI score, and histopathological evaluation

Safranin O is a basic stain that binds proteoglycans present in cartilage with a high affinity [15]. In brief, knee joints from rats were fixed for 72 h in 10% buffered formalin and decalcified for 3 weeks in 10% EDTA solution at 4 °C. Then, joints were embedded in paraffin blocks and cut into 5-μm-thick sections. Subsequently, knee joints were stained with Safranin O to evaluate cartilage destruction, which was graded using the Osteoarthritis Research Society International (OARSI) scoring system for medial tibial plateaus [16]. OARSI scores were graded on a scale of 0–6. All sections were stained in a single batch, and two experienced observers, who were blinded to the study, performed the scoring.

### COMP and CS846 biomarker analysis

Serum levels of COMP and CS846 of the rats in each group were determined using a sandwich enzyme-linked immunosorbent assay (ELISA) (catalog nos. EHJ-96099r and EHJ-96089r, Huijia Biological Technology Co., Ltd., Amoy, China). ELISA was performed according to the manufacturer’s instructions. A linear regression curve was drawn using the standards provided with the kit and was used to calculate the COMP and CS846 concentrations in each sample. All analyses were performed by the Heilongjiang Key Laboratory for Laboratory Animals and Comparative Medicine in Harbin.

### Statistics

SPSS software (version 19.0 for Windows, SPSS, Chicago, IL, USA) was used for all statistical analyses. Values are expressed as the mean ± SD. One-way analysis of variance (ANOVA) analysis was used for statistical comparisons between multiple groups. Correlations were analyzed using Spearman’s rank correlation analyses. AUC of the receiver operating characteristic (ROC) curve was used to assess the predictive value of COMP, CS846, and the combination thereof for OA. Multivariate regression analysis was employed to establish the diagnostic mathematical model. Based on this model, the prediction value was calculated, followed by ROC curve analysis. *P* < 0.05 was considered statistically significant.

## Results

### Macroscopic observation

**Fig. 1:**
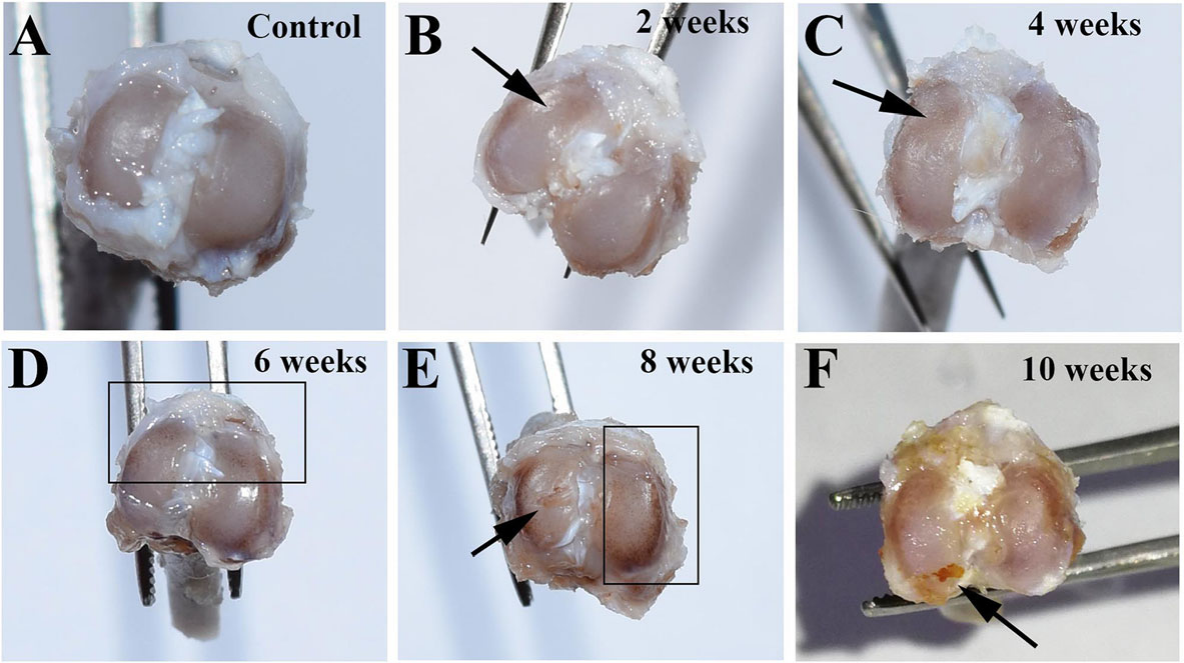
Macroscopic observation of tibial plateau. **a** Tibial plateau of rats in the control group showed a smooth cartilage surface, and no cartilage defects or osteophytes. **b** After 2 weeks of surgery, the joint surface was slightly rough (arrow). **c** The joint surface was partially recessed at 4 weeks of after surgery (arrow). **d** The joint surface was rough and matte at 6 weeks of after surgery (rectangle frame). **e** The articular cartilage was severely damaged at 8 weeks after surgery (rectangle frame). **f** The joint surface showed a large area of ulcers, and subchondral bone was exposed at 10 weeks after surgery (arrow)

**Fig. 2:**
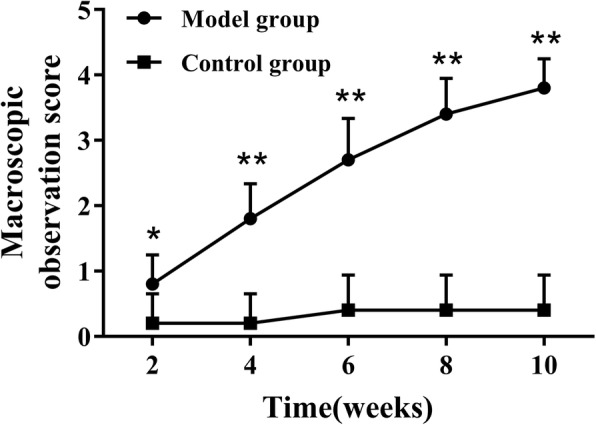
Macroscopic observation score of tibial plateau of rats in the model and control groups at various time points. Data are shown as the mean ± standard error of the mean. *P < 0.05 or **P < 0.01 compared with the control group

In the model group, all knees demonstrated cartilage degenerative changes of varied degrees (Fig. 1). Cartilage on the tibial plateau in the control group appeared macroscopically normal with a smooth surface, and no cartilage defects or osteophytes were observed (Fig. 1a). With the development of OA, the degree of articular surface ulceration in rats was increased and was gradually accompanied with a loss of luster. Two weeks after induction of OA, the joint surface had a slightly rough appearance (Fig. 1b). In addition, 4 weeks after induction of OA (Fig. 1c), the cartilage surface of ACLT rats was rough and uneven, the joint surface was dull, and local ulcers were formed. At 6 weeks after induction of OA, the middle layer of articular cartilage was eroded (Fig. 1d). Moreover, the articular cartilage surface was dark red in color and was severely damaged at 8 and 10 weeks after OA induction (Fig. 1e, f). In addition, the ulcerated area was increased, and the lower part of the cartilage was exposed. Accordingly, macroscopic observation (Fig. 2) showed that OA-related cartilage degradation increased with time after ACLT surgery. At each time point, the macroscopic observation scores in the model group were significantly higher when compared to those in the control group (*P* < 0.05).

### Histological analysis

**Fig. 3:**
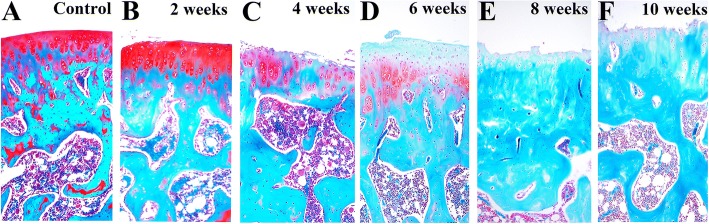
Representative safranin O-stained sections of articular cartilage. **a** In the control group, the cartilage surface was smooth and safranin O staining was uniform. **b** At 2 weeks after surgery, chondrocytes arranged at the surface area were uneven and the matrix was intact. **c** At 4 weeks after surgery, the intensity of proteoglycan staining decreased, whereas that of chondrocytes increased. **d** Irregular cartilage surface and chondrocyte disturbance were observed at 6 weeks after surgery. **e** Chondrocyte death and proteoglycan loss were observed at 8 weeks after surgery. **f** The extracellular matrix showed a loss in staining intensity; unequivocal dissipation of chondrocytes were observed at the lesion area at 10 weeks after surgery. Magnification × 10

**Fig. 4:**
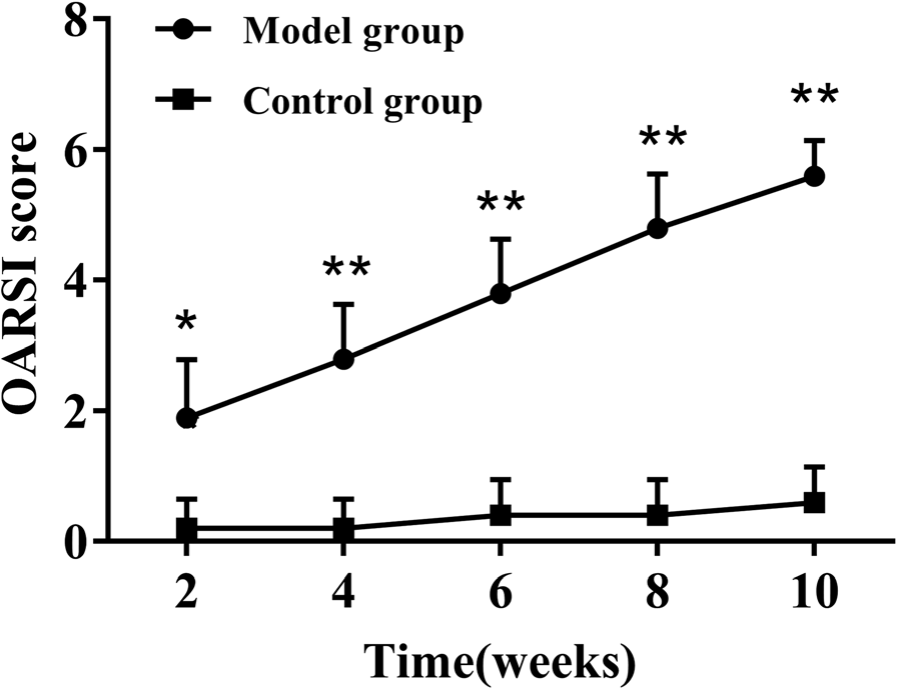
OARSI score of articular cartilage of rats in model and control groups at various time points. Data are shown as the mean ± standard error of the mean. *P < 0.05 or **P < 0.01 compared with rats in the control group

Figure 3 presents representative images of safranin O-stained rat joint tissues at 2, 4, 6, 8, and 10 weeks after ACLT surgery. In the control group, the articular surface was smooth, safranin O was evenly distributed, and chondrocytes were arranged in a regular manner (Fig. 3a). The different stages of OA in the model group showed different degrees of articular cartilage pathology. Two weeks after induction of OA, the surface of articular cartilage was partially rough, the matrix was intact, and chondrocytes in the superficial zone were arranged unevenly (Fig. 3b). At 4 weeks after surgery, the surface of articular cartilage was rough, staining intensity of the matrix was reduced, chondrocytes in the middle area were disordered, and chondrocytes were enlarged in size (Fig. 3c). In addition, at 6 weeks after surgery, surface irregularity, a decrease in safranin O staining, and disordered chondrocytes were observed (Fig. 3d). Knee joints from rats in the model group at 8 weeks after OA induction showed chondrocyte death, loss of the intercellular matrix, and thinning or destruction of the cartilage layer (Fig. 3e). Additionally, the matrix showed a loss of staining, and an unequivocal dissipation of chondrocytes was observed at the lesion area at 10 weeks after surgery (Fig. 3f). The OARSI scores of rats in the model group increased with the progression of OA. The OARSI score for cartilage degeneration showed a significant higher score in rats in the model group when compared to rats in the control groups (*P* < 0.05) (Fig. 4).

### COMP and CS846 levels

**Fig. 5:**
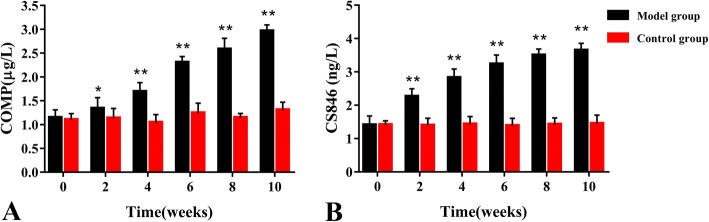
Serum biomarker levels of rats in the model and control groups at various time points. **a** Serum COMP concentration (μg/L). **b** Serum CS846 concentration (ng/L). Data are shown as the mean ± standard error of the mean. *P < 0.05 or **P < 0.01 compared with the control group

**Fig. 6:**
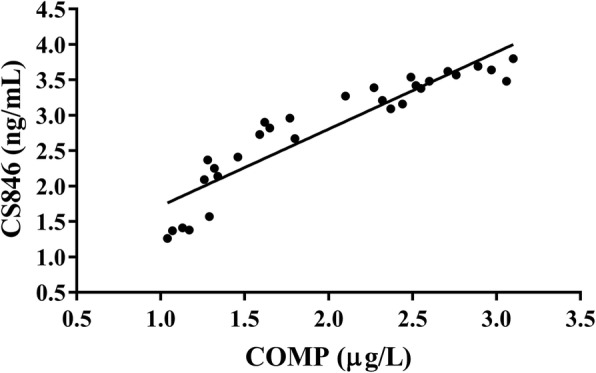
Correlation between serum levels of COMP and CS846 (*r* = 0.879)

The concentration of COMP and CS846 in knee joints of rats of the control and model groups at various time points are summarized in Fig. 5a, b. The levels of COMP and CS846 continuously increased over 10 weeks after ACLT surgery (COMP standard linear regression curve *r*^2^ = 0.99106; CS846 standard linear regression curve *r*^2^ = 0.99350). Compared with the control joint, COMP and CS846 levels significantly increased from the second to the tenth week after OA induction (*P* < 0.05). In addition, a positive correlation was observed between serum COMP levels and serum CS846 levels (*r* = 0.879, *P* < 0.001) (Fig. 6).

### Correlation between COMP and CS846 concentration and the OARSI score

**Fig. 7:**
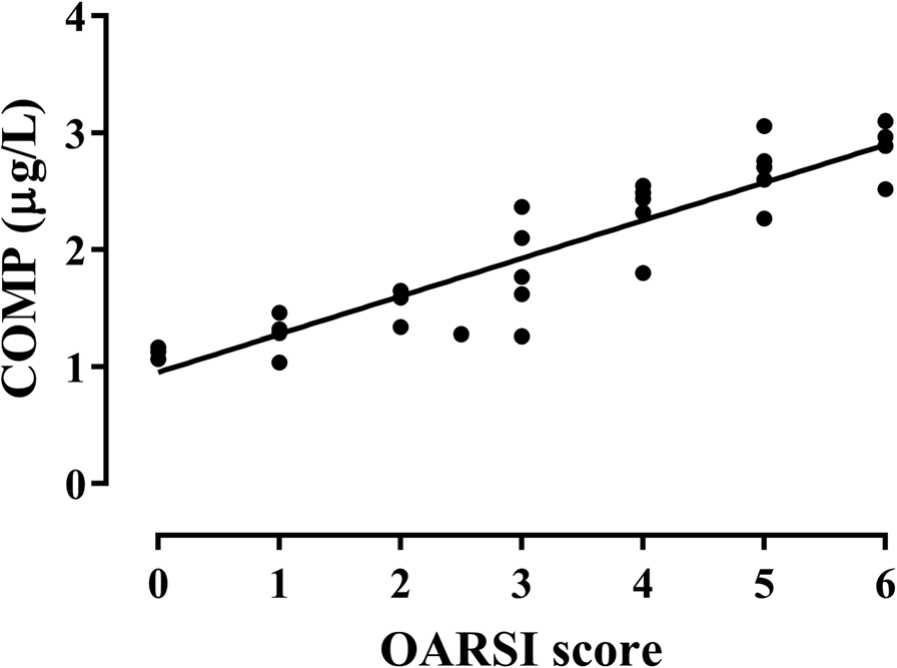
Correlation between serum levels of COMP and the OARSI score of the corresponding articular cartilage (*r* = 0.915)

**Fig. 8:**
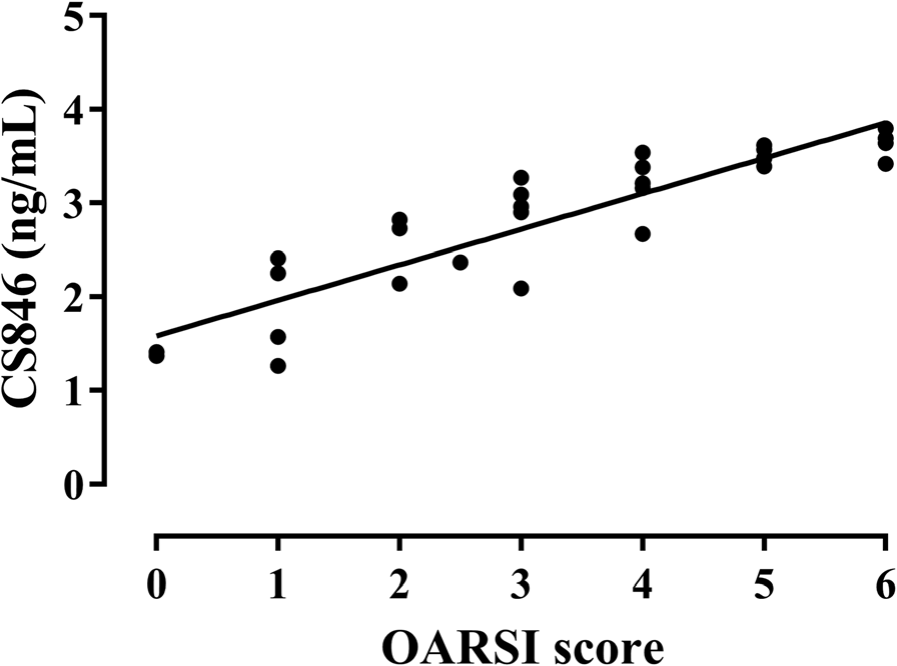
Correlation between serum levels of CS846 and the OARSI score of corresponding articular cartilage (*r* = 0.912)

A positive correlation was observed between changes in rat serum COMP levels and corresponding articular cartilage OARSI scores (*r* = 0.915, *P* < 0.001) (Fig. 7). In addition, a positive correlation was observed between changes in rat serum CS846 levels and corresponding articular cartilage OARSI scores (*r* = 0.912, *P* < 0.001) (Fig. 8).

### ROC curve analysis of single and combined biomarkers

**Fig. 9:**
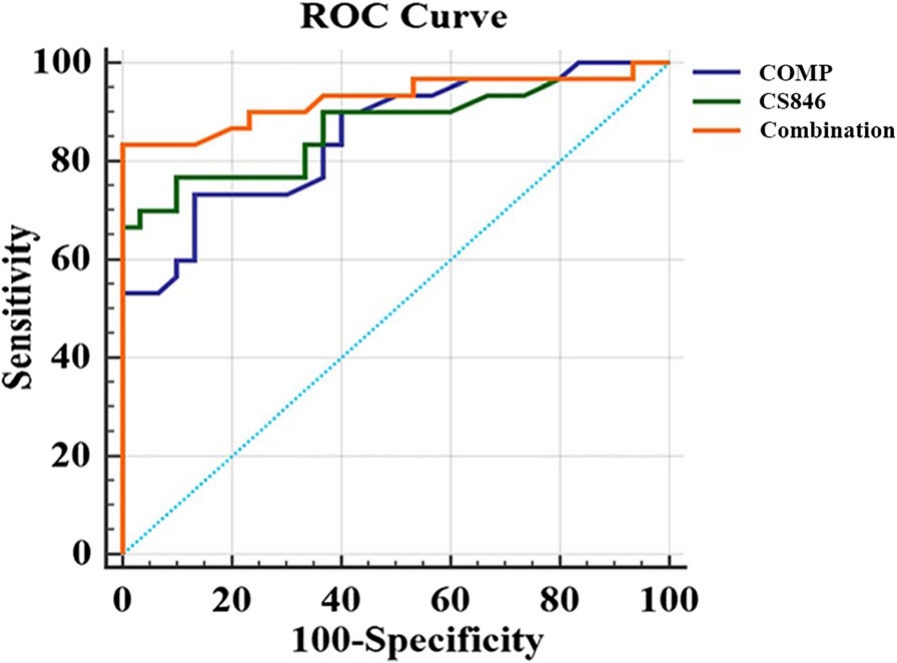
ROC curve analysis of single and combined biomarkers in OA prediction. The diagonal segment is the reference line

The ROC curve diagram showed that the area of COMP, CS846 and the combination thereof were > 0.5 (above the reference line), respectively, indicating that both single detection and combined detection were important for the diagnosis of OA (Fig. 9). Among them, the AUC of serum COMP, CS846, and combined biomarkers was 0.851, 0.868, and 0.926, respectively. Among all, the combined biomarkers yielded an ROC value, which was significantly higher when compared to that of single biomarkers (*P* < 0.05) and would be ideal for the diagnosis of OA.

## Discussion

In this study, we combined the expression of serum levels of COMP and CS846 in OA rats to provide a reference for early diagnostic criteria of OA. A sensitive and stable biochemical indicator was established. In the past few years, the importance of serum biomarkers in OA, synovitis, joint effusion, and the progression of cartilage damage has become increasingly appreciated and has been proven helpful for monitoring disease progression and clinical therapy [1, 17]. However, studies focusing on the magnitude and timing of the effects of OA on serum marker levels are limited. To our knowledge, this is the first study in which the value of combined detection of serum levels of CS846 and COMP was evaluated in early OA diagnosis. In addition, the ACLT model, in which permanent instability in the articular cartilage knee joint occurs, is a frequently used and well-accepted OA model [15, 18]. Thus, the rat OA model used in this study is an excellent translational model for estimating intra-articular inflammation with outcomes that are relevant to human OA [19].

Articular cartilage is composed of hyaline cartilage, which is composed of an abundant ECM and chondrocytes embedded therein, and with mechanical properties [2]. Degradation of the network of collagen and proteoglycans in OA cartilage leads to a loss in tensile strength and shear properties of cartilage [20]. COMP is a tissue-specific protein that binds to the type II collagen network. When articular cartilage is destroyed, COMP is released into the SF and subsequently absorbed in the serum [21]. COMP has shown promising effects as a prognosticator of rapid joint destruction in humans with levels in both serum and SF being increased in patients with OA when compared to those without joint disease [22]. Interestingly, Geng et al. reported that COMP deficiency enhanced the early onset and development of chronic arthritis but does not affect collagen II autoimmunity [23]. In addition, in a study performed by Fernandes et al., it was suggested that COMP could be used as a diagnostic marker for early OA as shown by elevated serum COMP levels in patients with knee pain symptoms without any radiological abnormalities, thereby indicating early cartilage damage in patients when compared to healthy controls [24]. Moreover, in a study by Huebner et al., SF levels of keratin sulfate (KS) and COMP increased, which was coincident with histological OA, and correlated positively with the severity of histological damage in guinea pig [25]. In our study, we showed that at 2 weeks after ACLT surgery, rats in the model group had significantly higher serum levels of COMP when compared to rats in the control group that at 4 weeks after surgery, they started to rapidly increase. In addition, at other time points, serum COMP levels of rats in the OA group were also higher when compared to those in the control group. In a previous study, similar results were obtained in rabbits with ACLT-induced OA [26]. These results indicated that serum COMP levels in rats in the model group increased early in OA development and gradually increased within 10 weeks after ACLT surgery. The increased release of COMP into serum during early OA is likely due to series of catabolic events that occur in articular cartilage, resulting in a high turnover rate by chondrocytes to repair the cartilage matrix. This process first led to dismantling of the cartilage matrix and subsequently a net loss of tissue. This inference is supported by our histological results, which indicated minor damage in the articular cartilage in rats in the model group at week 2 after surgery. Similar results were obtained by Bin et al. [27]. Also, the increase in SF COMP levels correlated with the length of time post injury, suggesting that COMP may be a suitable marker for longitudinal studies to evaluate its role in joint healing [28]. Georgiev et al. observed higher serum COMP levels in knee OA patients when compared to controls, and COMP correlated positively with Whole-Organ MRI Score, suggesting that COMP may reflect structural damage of the knee joint [29].

CS846 is a synthetic CS marker and inseparable from the degree of joint injury in patients with OA [10]. CS846 levels were found to be significantly increased in serum and SF of experimental equine OA models when compared to healthy controls, and levels further increased in response to exercise [30]. Levels of CS and CS846 were significantly increased in patients with juvenile idiopathic arthritis [31]. Previous studies have indicated that CS846 was almost absent from mature adult cartilage; however, CS846 is present at increased levels following OA [32, 33] and joint injury [34]. In a previous study, Svoboda et al. reported that changes in serum levels of CS846 from baseline to follow-up were significantly different between ACL-injured patients and uninjured controls [35]. In our study, we found that at all time points after surgery, serum levels of CS846 in rats in the model group were higher when compared to those in the control group. In addition, we found that CS846 serum levels increased significantly at 2 weeks after ACLT surgery. This may explain the degradation of proteoglycans observed in the early onset of OA. These findings were supported by our macroscopic observation and histological results, indicating that there was minor damage of articular cartilage in the model group at 2 weeks after surgery. Nevertheless, in the present study, post injury changes in CS846 between ACL-injured cases and uninjured controls were comparable with those observed between OA patients and healthy controls as reported by Svoboda et al. [35]. In previous studies, it has been suggested that the combination of urinary CTX-II, serum COMP, and serum CS846 levels is indicative of the amount of joint damage in patients with hemophilic arthropathy [36]. Based on the data obtained from prior studies and ours, we hypothesized that CS846 can be used as an OA biomarker to assess the extent of articular cartilage damage. In addition, we found that serum levels of CS846 and COMP increased with the development of OA, and the peak value was not detected at 10 weeks after ACLT surgery.

As the sensitivity of a single serum OA biomarkers in predicting OA was low, thereby limiting its potential in clinical application. Therefore, we analyzed the sensitivity when these markers were combined and obtained the AUC of ROC curve. We then calculated their diagnostic value in OA. The combined biomarkers yielded a ROC value of 0.926, which was significantly higher when compared to that of the single biomarker and can be better used for evaluation and diagnosis of OA. In previous studies, early detection of OA has been reported by a combination of biomarkers [26, 27, 37]. Nowadays, the search for early diagnostic markers of OA has become a hot topic. To investigate the correlation between levels of COMP and CS846 and the severity of articular cartilage degeneration, histological changes in articular cartilage at various time points were evaluated based on the OARSI scoring system. In this study, the OARSI scores of rats in the model group were significantly higher regarding both OARSI scores and macroscopic observation scores when compared to the control group. There was a highly positive correlation between COMP and CS846 levels and the OARSI scores (*r* = 0.915, *r* = 0.912, *P* < 0.001). We found that the higher the levels of COMP and CS846 in the serum of ACLT rats, the more severe the degree of articular cartilage lesions. The correlation of COMP was higher compared to that of CS846. Histological examination showed that within 10 weeks after the induction of OA, the surface roughness of articular cartilage gradually increased and the chondrocytes first became hypertrophied and then decreased. In addition, chondrocyte disorganization gradually increased, the intensity of safranin O staining decreased, and the matrix gradually degraded. At 10 weeks after surgery, the stroma showed almost no staining, and clear dissociation of chondrocytes was observed in the lesion area. Histology showed that at 2 weeks after ACLT surgery, the joint surface was rough and after 4 weeks, the degree of joint unevenness was aggravated. Similarly, macroscopic observations also demonstrated that the articular surface was rough at 2 weeks after surgery, and depression appeared on the articular surface at 4 weeks after ACLT surgery, followed by gradual increase of joint surface ulceration and destruction. In the ACLT model used in this study, articular cartilage degeneration was a slow and gradual process, which was in accordance with the true histological processes of OA. These findings indicated that the OA model established in this study was successful. Moreover, we found a positive correlation between changes in levels of COMP and CS846 in rat serum over the course of 10 weeks. As a biomarker, the serum concentration of COMP can reflect the degree of denaturation of type II collagen, whereas serum levels of CS846 can reflect the degree of proteoglycan degradation [35]. Together, these results showed that the degradation of both collagen and proteoglycans resulted in loss of the ECM, thereby causing progressive degeneration of articular cartilage. Synovial hyperplasia, fibrosis, secretion of inflammatory mediators, and cartilage degrading enzymes can lead to acceleration of OA, which intensifies the degradation of proteoglycans and the presence of aggrecan fragments bearing the CS846 epitope. The destruction of cartilage further degrades the collagen network and exacerbates the release of COMP into the SF, which is absorbed by the blood. ECM degradation of chondrocytes is a result of the combined action of type II collagen and proteoglycans [38]. The combined detection of COMP and CS846 fully reflects the degeneration of cartilage. This also indicated that the two indicators of early lesions in response to OA can be used as joint biomarkers for the diagnosis of OA. These results supported the idea that a combination of biomarkers may relate significantly better to the severity of joint damage than individual biomarkers do.

A single test for CS846 or COMP only partially reflected ECM degradation. Combined detection of serum CS846 and COMP concentrations predicted overall degradation of type II collagen and proteoglycans, thereby assessing the degree of OA and early diagnosis of OA. Although the results are promising, our study also has some limitations. Both macroscopic observational histological tests at 10 weeks after OA induction in rats in the model group showed that the degree of OA was significant and that the longitudinal study of 10 weeks was not sufficient. Accordingly, the peak value of CS846 and COMP levels in serum could not be determined at the end of this study. We only performed histological analysis of the tibial plateau, not including the femoral condyle. However, the results were reliable because OA caused articular cartilage degradation in the tibial plateau within 10 weeks after ACLT surgery.

## Conclusions

This study provides valuable information on the relationship between serum levels of COMP and CS846, and the degree of OA. Within 10 weeks after ACLT, levels of COMP and CS846 increased significantly, and a positive correlation was observed between the two biomarkers. Serum levels of COMP and CS846 positively correlated with the OARSI score during the OA progression. The combined biomarkers yielded a ROC value, which was significantly higher when compared to that of the single biomarker and would be better if used for the diagnosis of OA. In conclusion, combined detection of serum levels of COMP and CS846 has potential in assessing the degree of OA and OA diagnosis.

## Abbreviations

ACL: Anterior cruciate ligament
ACLT: Anterior cruciate ligament transection
COMP: Cartilage oligomeric matrix protein
CS: Chondroitin sulfate
CS846: Chondroitin sulfate 846 epitope
ECM: Extracellular matrix
GAG: Glycosaminoglycan
KS: Keratin sulfate
OA: Osteoarthritis
SF: Synovial fluid
